# RETRACTED: Sabeela et al. Reactive Mesoporous pH-Sensitive Amino-Functionalized Silica Nanoparticles for Efficient Removal of Coomassie Blue Dye. *Nanomaterials* 2019, *9*, 1721

**DOI:** 10.3390/nano15201540

**Published:** 2025-10-10

**Authors:** Nourah I. Sabeela, Tahani M. Almutairi, Hamad A. Al-Lohedan, Abdelrahman O. Ezzat, Ayman M. Atta

**Affiliations:** 1 Surfactants Research Chair, Chemistry Department, College of Science, King Saud University, Riyadh 11451, Saudi Arabia; noonah-37@hotmail.com (N.I.S.); hlohedan@ksu.edu.sa (H.A.A.-L.); ao_ezzat@yahoo.com (A.O.E.); 2 Chemistry Department, College of Science, King Saud University, Riyadh 11451, Saudi Arabia; talmutarai@ksu.edu.sa

The journal retracts the article “Reactive Mesoporous pH-Sensitive Amino-Functionalized Silica Nanoparticles for Efficient Removal of Coomassie Blue Dye” [1], cited above.

Following publication, concerns were brought to the attention of the publisher regarding the reliability of Figure 3 presented in this article [1].

Adhering to our complaint procedure, an investigation was conducted, which revealed multiple irregularities in Figure 3. While the authors cooperated with the Editorial Office during the investigation, they were unable to satisfactorily explain the irregularities present in this image, nor provide the appropriate raw material for Editorial Board evaluation.

As a result, the Editorial Board has lost confidence in the reliability of the findings and has decided to retract the publication [1], as per MDPI’s retraction policy:

https://www.mdpi.com/ethics#_bookmark30

This retraction was approved by the Editor-in-Chief of the journal *Nanomaterials*.

The authors did not agree to this retraction.
